# Netrin-1 overexpression is predictive of ovarian malignancies

**DOI:** 10.18632/oncotarget.258

**Published:** 2011-05-02

**Authors:** Anastasios D. Papanastasiou, Georgios Pampalakis, Dionyssios Katsaros, Georgia Sotiropoulou

**Affiliations:** ^1^ Department of Pharmacy, School of Health Sciences, University of Patras, Rion-Patras, 26500, Greece; ^2^ Department of Obstetrics and Gynecology, Gynecologic Oncology Unit, University of Turin, Turin, Italy

**Keywords:** Netrin-1 (NTN1), ovarian cancer, biomarker

## Abstract

Netrin-1 (NTN1) is functionally important for the development of the nervous system. Interestingly, few recent studies showed that NTN1 may also promote cancer by increasing survival and resistance of lung and breast cancer cells to apoptosis. Our purpose was to investigate whether NTN1 and its receptor DCC may be involved in ovarian cancer. The *NTN1* and *DCC* mRNAs were quantified by real-time RT-PCR in normal (10), benign (8) and cancer (17) ovarian tissues. *ALAS1* and *TBP* housekeeping genes were used for normalization. *NTN1* was found overexpressed in 76% of ovarian cancer specimens (13/17) as compared to normal (0/10, p<0.004) and benign (1/8, p<0.008) samples. Increased *NTN1* mRNA levels correlated with advanced tumor stage (stage III, n = 8, 100%) and grade (grade 3, n = 7, 100%). In contrast, *DCC* was found downregulated in 59% (10/17) of ovarian tumors tested but correlation was not significant when compared to normal or benign specimens. Here, we demonstrated that NTN1 may be involved in ovarian cancer as the expression of *NTN1* mRNA is strongly upregulated in ovarian malignant tumors but not in benign tumors. The fact that increased *NTN1* is specifically observed in cancerous tissues indicates that *NTN1* may represent a novel candidate biomarker for ovarian cancer.

## INTRODUCTION

Netrin-1 (NTN1) is a diffusible laminin-related protein that has been shown to play a major role in the control of neural navigation in the developing nervous system [1, 2]. Recently, NTN1 was proposed to play crucial roles in tumorigenesis as it is involved in regulation of apoptosis by binding to DCC and UNC5H family of dependence receptors that share the ability to induce apoptosis in the absence of their ligands, a trait that confers tumor suppressor activity on these receptors [3]. Expression of one of these dependence receptors at the surface of a tumor cell is considered to render it dependent on ligand availability for survival, thus, inhibiting uncontrolled tumor cell proliferation and/or metastasis. Accumulating evidence implies that this positive signaling pathway is frequently inactivated in human cancers, as it was shown that expression of DCC and UNC5H is lost during tumor progression, thus, conferring a selective advantage to the tumor cell for survival [3]. In addition, this model predicts that a similar advantage may be obtained by gaining autocrine expression of the NTN1 ligand in tumor cells. Indeed, a high percentage of colorectal [4] and metastatic breast [5] cancers overexpress *NTN1*.

Binding of NTN1 to its receptors inhibits p53-dependent apoptosis, while p53 is directly involved in transcriptional regulation of *NTN1* and its receptors [3]. Thus, *NTN1* expression confers resistance to apoptosis, thus, enhancing tumor cell survival. In non-small cell lung cancer (NSCLC), >50% of the analyzed tissue specimens expressed abnormally high levels of *NTN1* [6, 7]. Interestingly, a recent study revealed that *NTN1* expression is selectively up-regulated in colorectal cancers associated with bowel diseases. Moreover, it was demonstrated that this inflammation-driven *NTN1* up-regulation is causal for colorectal cancer development, as interference with the *NTN1* autocrine loop inhibited colorectal cancer progression without affecting inflammation in a mouse model for ulcerative colitis-associated colorectal cancer [4].

In this direction, it was shown recently that autocrine production of NTN1 in aggressive neuroblastomas conveys selective advantage for tumor growth and dissemination [8], as it blocks the pro-apoptotic activity of the NTN1 receptors, UNC5H, leading to resistance to apoptosis that is a prerequisite for neuroblastoma progression. *NTN1* up-regulation was suggested as a potential marker for poor prognosis in infants diagnosed with stage 4S neuroblastoma [8]. It was proposed that interference with the NTN1 autocrine loop in malignant neuroblasts may represent an alternative therapeutic strategy, as it was shown that disruption of this loop triggers cell death and inhibits metastasis of neuroblastoma in avian and mouse models [8].

Interestingly, NTN1 was proposed to play an important role in embryonic and pathological angiogenesis acting as a survival factor also for endothelial cells by blocking the pro-apoptotic effect of the dependence receptor UNC5B and its downstream death-signaling effector, the serine/threonine kinase DAPK. Thus, the proapoptotic effect of unbound UNC5B and the survival effect of NTN1 on endothelial cells finely tune the angiogenic process [9]. Nonetheless, in a recent study, *NTN1* was found strongly up-regulated in pancreatic adenocarcinomas and this aberrant *NTN1* overexpression was associated with resistance to apoptosis for both tumor and endothelial cells [10]. Triggered by these recent studies pinpointing to important roles of NTN1 in most common malignancies, we investigated here whether *NTN1* expression changes between normal, benign and cancerous ovarian tissues. In addition, we examined the expression of the NTN1 receptor *DCC*. It must be noted that DCC is considered a tumor suppressor that was originally identified based on allelic deletions at 18q21 associated with colorectal cancer [11].

## RESULTS AND DISCUSSION

**Figure 1:**
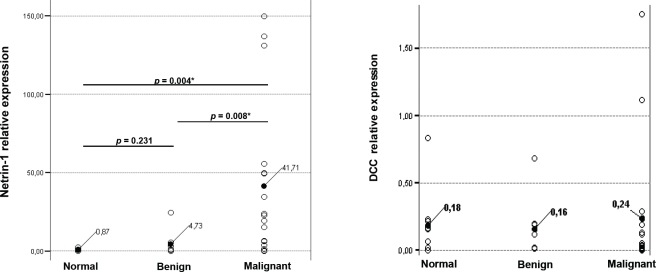
NTN1 and DCC mRNA expression in ovarian samples Relative expression levels of *NTN1* (left) and *DCC* (right) were analyzed in the clinical samples. Changes between benign-normal and cancer-normal are considered significant with both the T-test and Mann-Whitney U test (shown are p values calculated by the T-test).

As shown in Figure 1 and Table 1, strong upregulation of *NTN1* expression was detected in the majority of ovarian cancer specimens when compared to normal (p<0.004) or benign (p<0.008) which is significant according to the T-test values. Expression was considered high when the ratio NTN1/reporter genes (ALAS1-TBP) was ≥6 and for DCC/reporter genes (ALAS1-TBP) ≥0.1. More importantly, upregulation of *NTN1* mRNA correlated well with advanced stage of disease (p<0.005, Mann-Whitney U test). It should be noted that a larger cohort of samples should be analyzed, in order to interrogate whether *NTN1* expression can be used to predict disease-free survival and/or overall survival. Contrary, *DCC* was found downregulated in 10/17 (59%) cancer specimens, which is well in accordance with previous observations that reported decreased DCC in 55% of ovarian cancers when measured by immunohistochemistry and RT-PCR in a much larger number of patients [14, 15]. Interestingy, re-expression of DCC in ovarian cancer cells induced an apoptosis phenotype [15]. DCC deactivation results from loss-of-heterozygosity in a subset of ovarian cancers [16]. We were unable to correlate the decreased *DCC* expression to tumor grade or stage probably due to the small number of samples analyzed. Nonetheless, it should be noted that 7/17 cancer specimens exhibited high DCC expression which correlated with *NTN1* upregulation in 6/7 samples (p<0.098 Pearson correlation). Expression of *DCC* in this subset of ovarian cancers is counterbalanced by increased *NTN1* levels, thus, again leading to evasion of apoptosis. When all 35 samples (normal, benign and cancer) were included in the analysis, no correlation (p<0.519) was derived between *NTN1* and *DCC* expression.

**Table 1:** Clinicopathological features for patient samples correlated to *NTN1* and *DCC* expression in ovarian tissue specimens

			*NTN1*			*DCC*		
		Cases	High expression	Low expression	*p* value	High expression	Low expression	*p* value
Normal		10	0/10 (0%)	10/10 (100%)		6/10 (60%)	4/10 (40%)	
Age	≥54	4	0/4 (0%)	4/4 (100%)		3/4 (75%)	1/4 (25%)	
	<54	6	0/6 (0%)	6/6 (100%)		3/6 (50%)	3/6 (50%)	
Benign		8	1/8 (12.5)	7/8 (87.5)		5/8 (62.5%)	3/8 (37.5%)	
			relative to normal		0.231			0.829
Age	≥54	2	0/2 (0%)	2/2 (100%)		1/2 (50%)	1/2 (50%)	
	<54	6	1/6 (17%)	5/6 (83%)		4/6 (67%)	2/6 (33%)	
Tumor		17	13/17 (76%)	4/17 (24%)		7/17 (41%)	10/17 (59%)	
			relative to normal		0.004^*^			0.675
			relative to benign		0.008^*^			0.977
Age	≥54	10	8/10 (80%)	2/10 (20%)		5/10 (50%)	5/10 (50%)	
	<54	7	4/7 (57%)	3/7 (43%)		2/7 (28.6%)	5/7 (71.4%)	
Grade	0	1	1/1 (100%)	0/1 (0%)		1/1 (100%)	0/1 (0%)	
	1	1	0/1 (0%)	1/1 (100%)		1/1 (100%)	0/1 (0%)	
	2	5	3/5 (60%)	2/5 (40%)		1/5 (20%)	4/5 (80%)	
	3	7	7/7 (100%)	0/7 (0%)		4/7 (57%)	3/7 (43%)	
			relative to grade ≤2		0.013^**^			0.414
	U	3	2/3 (67%)	1/3 (33%)		0/3 (0%)	3/3 (100%)	
Stage	I	5	2/5 (40%)	3/5 (60%)		1/5 (20%)	4/5 (80%)	
	II	1	1/1 (100%)	0/1 (0%)		1/1 (100%)	0/1 (0%)	
	III	8	8/8 (100%)	0/8 (0%)		5/8 (62.5%)	3/8 (37.5%)	
			relative to stage ≤2		0.005^**^			0.805
	U	3	2/3 (67%)	1/3 (33%)		0/3 (0%)	3/3 (100%)	
High expression is considered the ratio NTN1/reporter genes (ALAS1-TBP) ≥ 6 and DCC/reporter genes (ALAS1-TBP)≥ 0.1								
U: Unknown characteristics								
^*^statistically significant, T-test values								
^**^statistically significant, Mann-Whitney U test								

In summary, we show here for the first time that *NTN1* is aberrantly overexpressed in the vast majority of malignant ovarian tumors but not in benign tumors. Moreover, high *NTN1* expression correlates well with both tumor stage and grade. The observed alteration in *NTN1* expression upon progression to malignancy can safely be considered significant as it pertained to 100% of malignant tumors, while in normal and benign tissues *NTN1* was barely expressed. Lack of expression in normal/benign is a desired but rare feature of cancer biomarkers. Our finding that *NTN1* expression can possibly be used as a biomarker to distinguish benign from malignant ovarian tumors, also implies that NTN1 may be implicated in the progression of benign disease to malignancy. Our improved understanding of the mechanisms underlying the key emergent property of cancers, their malignancy, is expected to promote our efforts to reduce cancer mortality [17]. Based on the intriguing finding that *NTN1* expression is not altered in benign ovarian tumors, we propose that *NTN1* expression should be evaluated in prospective studies as a promising candidate biomarker to distinguish benign from malignant tumors of the ovary.

## PATIENTS AND METHODS

### Clinical samples

All clinical samples used in this study were collected at the University of Turin, Italy. Tissues were obtained following informed and signed consent of the patient under a general tissue collection protocol approved by the Institutional Review Board and the University of Turin, Italy. The samples were snap-frozen in liquid nitrogen and stored at −80°C.

### RNA extraction

RNA extraction was performed with RNeasy (Qiagen) after tissue pulverization with a pestle and mortar under liquid nitrogen. RNA purity and integrity were confirmed by spectrophotometry and agarose gel electrophoresis, respectively.

### Reverse Transcription and Quantitative PCR

500 ng of total RNA were reverse-transcribed with Superscript II (Invitrogen) using an oligo dT primer according to the standard protocol. The total volume of the recovered cDNA was 21 μl. Real-time PCR was carried out in 25 μl with SYBR Green (Invitrogen) in a Rotor Gene 3000 (Cobert Research). The following conditions were applied for PCR amplification: 50°C for 2 min, 94°C for 5 min followed by 45 cycles (40 for *ALAS1*, *TBP*) of 94°C for 15 sec, 54°C for 20 sec (54°C for 30 sec for ALAS1, TBP), and 72°C for 20 sec (30 sec for *ALAS1*, *TBP*). The sequences of *NTN1* primers were: 5'-TGC AAG AAG GAC TAT GCC GT-3' and 5'-GCT CGT GCC CTG CTT ATA CAC-3' and for DCC were: AGC CAA TGG GAA AAT TAC TGC TTA C and AGG TTG AGA TCC ATG ATT TGA TGA G [8]. *ALAS1* and *TBP* primer sets for real-time PCR were obtained from Qiagen (QuantiTect Primer Assay). Serial dilutions of cDNAs from selected tissue samples were used to determine the amplification efficiency of *NTN1*, *DCC*, *ALAS1* and *TBP*. The Pfaffl model [12] and the REST 2009© relative expression software tool [13] were used to determine the amplification efficiency and estimate the relative changes in mRNA levels of *NTN1* and *DCC*. Data normalization was carried out against both *ALAS1* and *TBP1* housekeeping genes, for improved reliability of results. Efficiency and fold-change were calculated based on cycle-threshold values using the REST software and *ALAS1* and *TBP1* as controls. All samples were analyzed in triplicates in two independent experiments.

### Classical RT-PCR and sequencing

Finally, semiquantitative conventional RT-PCR was performed with OneStep (Qiagen) and the PCR products were resolved by agarose gel electrophoresis (not shown). Two randomly selected products were gel-extracted (Qiagen) and sequenced using the *NTN1*-specific forward primer (VBC, Austria), in order to verify that the PCR product was indeed *NTN1*-specific.

### Statistical analysis

The Mann-Whitney U test or T-test were used to correlate clinical parameters (stage, grade) and *NTN1* or *DCC* relative mRNA expression levels. Statistical analyses were performed with SPSS (SPSS® release 15.0, Chicago, IL, USA).
